# Mathematical processing of absorption as green smart spectrophotometric methods for concurrent assay of hepatitis C antiviral drugs, Sofosbuvir and Simeprevir: application to combined pharmaceutical dosage forms and evaluation of the method greenness

**DOI:** 10.1186/s13065-023-00984-5

**Published:** 2023-07-14

**Authors:** Sayed M. Derayea, Ahmed A. Abu-hassan, Ahmed A. Hamad, Walid E. Eltoukhi, Amal E. Hamad, Bassam Shaaban Mohammed

**Affiliations:** 1 Department of Analytical Chemistry, Faculty of Pharmacy, Minia University, Minia, 61519 Egypt; 2 Pharmaceutical Analytical Chemistry Department, Faculty of Pharmacy, Al-Azhar University, Assiut Branch, Assiut, 71524 Egypt; 3 Department of Pharmaceutical Analytical Chemistry, Faculty of Pharmacy, Menoufia University, Shebin El‐Kom, Menoufia Egypt

**Keywords:** Simeprevir, Sofosbuvir, Iso-absorptive point method, Ratio subtraction method, Dual wavelength method, Combined pharmaceutical dosage forms

## Abstract

The present work was developed to create three rapid, simple, eco-friendly, cheap spectrophotometric methods for concurrent assay of Sofosbuvir (SOF) and Simeprevir (SMV) in their pure, laboratory prepared mixture and pharmaceutical dosage form with high degree of accuracy and precision. Three methods were developed including iso-absorptive point, ratio subtraction and dual wavelength. The linear range of the proposed methods was 3.0–50.0 and 2.0–50.0 µg mL^−1^ for SMV and SOF, respectively. The proposed methods were validated according to ICH guidelines in terms of linearity, accuracy, precision, limit of detection and limit of quantitation. The proposed approach is highly simple and the procedure is environmentally green making it suitable for the drug analysis in routine works.

**Supplementary Information:**

The online version contains supplementary material available at 10.1186/s13065-023-00984-5.

## Introduction

Hepatitis C virus is a major culprit of end-stage liver disease that end up requiring liver transplantation, and millions of people around the world are affected by this serious infection. The introduction of direct-acting oral antiviral agents (DAAs) that effectively disrupts HCV replication led to a dramatic improvement in the treatment of this debilitating disease. They offered simpler treatment regimen with less side effects and even higher efficacy, compared to older regiments involving interferons, making them currently the first line of treatment [1–8]. Combination regimens soon followed for more complicated conditions or simply for patients opting for an all-oral treatment. SMV-SOF was the first of such combinations.

**Fig. 1:**
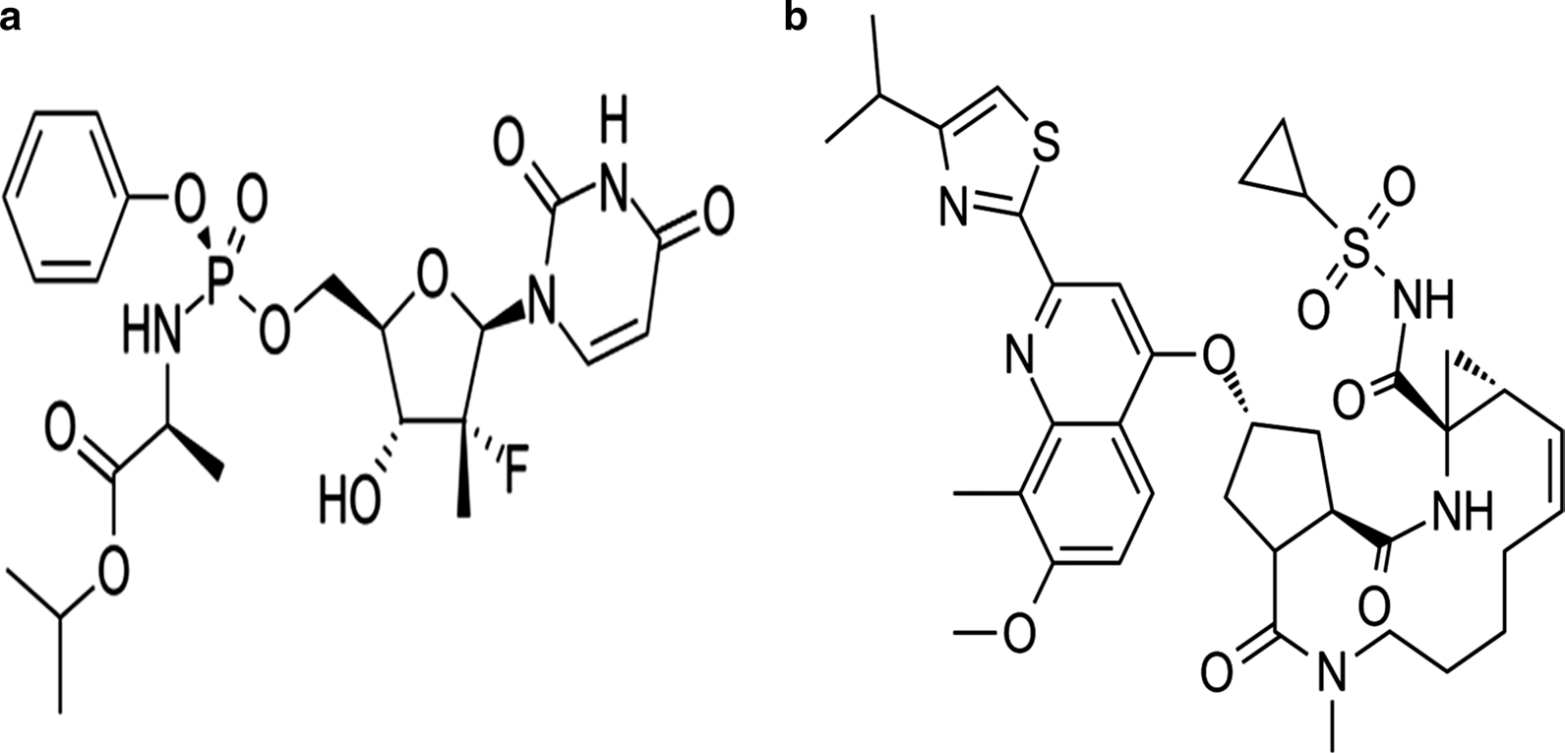
**a**: Chemical structures of Sofosbuvir (SOF). **b**: Chemical structures of Simeprevir (SMV)

Sofosbuvir (SOF), and Simeprevir (SMV) (Fig. 1) are two antiviral drugs that have been in clinical use for less than a decade thus yet attracted a lot of attention in the field of pharmaceutical analysis.

A recent review [9] described the reported analytical methods for the assessment and monitoring these drugs, alone or in combination, in raw material, dosage forms and biological fluids. It was clear that the majority were in the realm of chromatographic techniques, LC–MS/MS to be more specific, which offered selectivity, sensitivity and applicability to complex matrices. The structure elucidation of degradants of active pharmaceutical ingredient (API), impurities, or the fragment from an excipient from the drug is offered by LC MS/MS technique. Nevertheless, spectrophotometric and fluorimetric techniques managed to have a footing deriven by the need for simple, inexpensive techniques that are still sensitive enough and reliable to assess these drugs, especially in dosage forms.

Spectrophotometer is one of the green chemistry approaches in chemical analysis if proper conditions are carefully selected. However, a great overlap was observed in the spectra of SMV and SOF, thus chemometrics was opted in the present work to enable their simultaneous spectrophotometric determination. Chemometrics encompasses proven simple, fast, accurate and low cost techniques in comparison with other analytical techniques like LC MS/MS to resolve complex mixtures with overlapping spectra without resorting to pre-analysis separation [10]. They can be easily applied in both research and quality control laboratories without the need to the expensive equipment or special training or software. With a myriad of mathematical approaches on offer, the analyst has the chance to pick the one that best fits his needs.

## Principle and theoretical calculations:

### Method I: iso-absorptive point method [11]

As the name implies, this technique exploits equal absorptivity of two different entities at a certain wavelength (λ). This λ is called the iso-absorptive point (ISP), at which: A_1_^1%^_1 cm_ = A_2_^1%^_1 cm_ = A_ISP_^1%^_1 cm_, where A_ISP_^1%^_1 cm_ is the absorptivity of either of them at a concentration of 1.0 g/100 mL and a path length of 1 cm.

If there is a mixture of the two drugs, we can calculate the total concentration of both drugs (C_TM_) using the absorbance of the mixture at ISP as follows:A_M_ = A_ISP_^1%^_1 cm_ (C_1M_ + C_2M_) = A_1_^1%^_1 cm_ (C_TM_).

Where A_M_ is the mixture absorbance at the iso-absorptive point and C_1M_ and C_2M_ are the concentrations of drug 1 and 2 in the mixture, respectively. All that remains is to determine the concentration of one of them by another method to find the concentration of the other by subtraction.

### Method II: ratio subtraction method [11–13]

This method works well for a combination of two drugs A and B with overlapped spectra without the need of an ISP provided that one of them (B) has an extended part in its spectrum of zero order with no interference from drug A. The concentration of drug A can be determined using ratio subtraction where the zero-order spectrum of the mixture is divided by the zero-order spectrum of a certain concentration of B named as the divisor (B'). This results in a plateau (constant absorbance) in the extended region of drug (B).

$$\frac{{\left( {A\, + \,B} \right)}}{{B^{`} }}\, = \,\frac{A}{{B^{`} }}\, + \,\frac{B}{{B^{`} }}\, = \, \frac{A}{{B^{`} }}\, + \, constant$$

If this constant is subtracted and the resulting spectra is multiplied by the divisor (B') then the original spectrum of A will be isolated, and its concentration can be determined by applying the linear regression equation of its calibration at its λ_max_. Drug B can be directly determined from the absorbance at wavelength in the spectrum where A is not showing any absorbance.

### Method III: dual wavelength method [11, 14]

For this method to work, the overlapping spectra need to exhibit two wavelengths where one drug (B; interfering compound) shows equal absorbance (thus ∆A_B_ equal zero), whereas the other one (A; drug of interest) shows significant difference in absorbance (∆A_A_) that is directly proportional to its concentration while totally independent on B. This allows for the determination of A while B can be determined directly from the area of the spectrum free from A absorbance.

## Experimental

### Instrumentation

Acculab Single Beam UV Visible Spectrophotometer UVS-85 was used for all spectrophotometric measurements. Wavelength Range between 200 and 1050 nm and using Deuterium, Tungsten Halogen Lamp with Silicon Photodiode detector. Band width was 2 nm with Wavelength Accuracy ± 1.0 nm and Photometric Accuracy was ± 0.5%T. all measurements in 1.0 cm Quartz cuvettes, using UV-85 Professional software.

### Chemical and reagents

Throughout this work chemicals and reagents used were of analytical grade. SMV pure powder and its dosage form Merospevir^®^ capsules (BN 160117—150 mg SMV/capsule) were given as a gift from AUG Pharma (6th Industrial Zone, 6th October City, Egypt). SOF pure powder was obtained from Egyptian International Pharmaceutical Industry (EIPICO, 10th of Ramadan City, Egypt). Sofolanork^®^ tablets (batch number M1001017 containing 400 mg SOF/tablet) were from Mash Premiere for Pharmaceutical Industry (3rd industrial zone, Badr City, Egypt). Solvents were purchased from El-Nasr chemical Co., (Abo-Zaabal, Cairo, Egypt). Merospevir (SMV) capsules contain the following inactive ingredients: colloidal anhydrous silica, croscarmellose sodium, lactose monohydrate, magnesium stearate and sodium lauryl sulphate. The white capsule contains gelatin and titanium dioxide and is printed with ink containing iron oxide black and shellac. While sofolanork (SOF) tablet include the following: colloidal silicon dioxide, croscarmellose sodium, magnesium stearate, mannitol, and microcrystalline cellulose. The tablets are film-coated with a coating material containing the following inactive ingredients: polyethylene glycol, polyvinyl alcohol, talc, titanium dioxide, and yellow iron oxide.

### Standard solutions of SMV and SOF

Accurately weighed 10.0 mg of each drug’s pure powder were dissolved separately in 100 ml volumetric flasks using ethanol. The volume was completed to the mark giving a stock standard solution containing 100.0 µg mL^−1^. All solutions were refrigerated until needed.

### General methods of analysis

#### Construction of calibration graphs

For the purpose of SMV determination in all three methods, SMV calibration graph was developed through plotting the absorbances of series of its pure solutions (3.0–50.0 µg mL^−1^) at 335 nm against their corresponding concentration and its linear regression equation was established. The concentration of SMV was directly determined in all methods from the mixture’s absorbance at 335 nm through its calibration graph’s linear regression equation.

### Method I (iso-absorptive point)

Zero order spectra of SMV (20.0 µg mL^−1^), SOF (20.0 µg mL^−1^) and a mixture containing both drugs (10.0 µg mL^−1^ SMV and 10.0 µg mL^−1^ SOF) were recorded. SOF calibration curve was constructed by measuring the absorbance of a series of its standard solutions (2.0–50.0 µg mL^−1^) at 273 nm. To analyze mixtures containing the two drugs, their absorbances at both 273 nm and 335 nm were recorded. The total concentration of the drugs in the mixture was determined from the linear regression equation of SOF calibration curve. SMV concentration in the mixture was determined using the absorbance at 335 nm as described above, then SOF concentration was calculated by subtraction. The raw UV spectra from the program of the spectrophotometer to iso-absorptive point shown in (Additional file 1: Figure S1).

### Method II (ratio subtraction)

The calibration curve was constructed for SOF at its λ_max_ of 260, using series of its standard solutions (2.0–50.0 µg mL^−1^). SMV (20.0 μg mL^−1^) was chosen as the divisor and its spectrum was recorded (B'). The spectrum of the binary mixture of SOF and SMV (A + B) was also recorded then divided by B' giving the resulting spectrum (*Spec 1*) represents $\frac{{\varvec{S}}{\varvec{O}}{\varvec{F}}}{{\varvec{S}}{\varvec{M}}{\varvec{V}}}+{\varvec{c}}{\varvec{o}}{\varvec{n}}{\varvec{s}}{\varvec{t}}{\varvec{a}}{\varvec{n}}{\varvec{t}}$. *Spec 1*showed a constant absorbance between 325 and 345 nm, after subtracting the ratio spectrum of SOF/SMV (*Spec 2*) was obtained. A simple multiplication of the ratio spectrum (*Spec 2*) by B` resolved SOF original spectrum that was part of the mixture. The concentration of SOF can be determined from the resolved spectrum using the linear regression equation of its calibration graph.

### Method III (dual wavelength method)

For SOF calibration graph, its pure solutions (4.0–50.0 µg mL^−1^) absorbances at 261 nm and 294 nm were recorded. ΔA_SOF_ was calculated as A_261 nm_–A_294 nm_ and were plotted against their corresponding concentration. From this calibration graph, SOF linear regression equation was determined. The raw UV spectra from the program of the spectrophotometer to Dual wavelength method shown in (Additional file 1: Figure S2).

#### Analysis of laboratory prepared mixtures

Solutions with different ratios of the studied drugs (1:1, 1:2, 2:1, 1:3, 3:1, 2:3 and 3:2 of SMV and SOF respectively) were prepared by transferring accurate aliquots of SMV and SOF stock solutions (100.0 μg mL^−1^) into a series of 10 mL volumetric flasks. The solutions were completed to the mark with ethanol and mixed well. The general methods of analysis were then applied (“Construction of Calibration Graphs” section).

#### Application to pharmaceutical formulations

Ten Merospevir^®^ capsules were emptied, the contents of which were mixed well and accurately weighed. Ten Sofolanork^®^ tablets were finely powdered and accurately weighed. From each powder, an amount equivalent to 150.0 mg of SMV and 400.0 mg SOF) was weighed and transferred into 100 mL calibrated flask. For extraction of SMV and SOF, 50 mL of ethanol were added, and subjected to sonication for 5 min. More ethanol was added to reach the final volume and the contents of the flasks were mixed well before being filtered discarding the first portion. A portion of the filtrate was diluted with ethanol to reach a final solution that has 150.0/ 400.0 µg mL^−1^ of SMV/ SOF, respectively. Aliquots of this solution were further diluted with ethanol then follow the procedure of analysis of laboratory mixture to calculate the concentrations of the SMV and SOF.

## Results and discussion

**Fig. 2:**
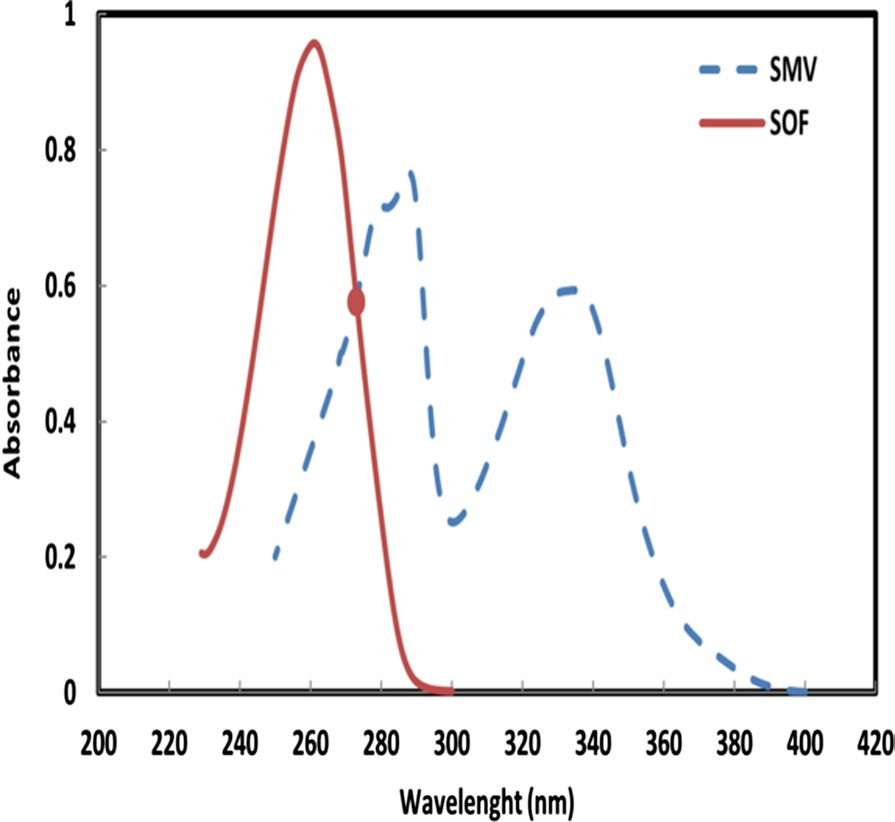
The absorption spectra of SMV 40.0 µg mL^−1^ and SOF 40.0 µg mL^−1^ showing the intersection point at 273 nm

Only 1 year after the approval of SOF and SMV individually, their combination was also approved, omitting the need for poorly tolerated interferon and achieving high cure rates in patients with and without cirrhosis [15, 16]. Although SMV can be easily determined in the presence of SOF, the opposite is not true. This is evident from their spectra which overlap throughout SOF absorption spectrum between 200 and 290 nm (Fig. 2). In this work, three chemometric methods were developed; iso-absorptive point, ratio subtraction and dual wavelength methods to address the problem of overlapping. The choice of chemometric methods was to offer simplicity, rapidity, affordability as well as reliability to analysts faced with the challenge of simultaneous determination of SOF and SMV in their bulk powders and pharmaceutical dosage form. No prior separation was required, just simple mathematical manipulations of the investigated drugs’ spectra that doesn’t require special equipment or extensive training and could be reliably applied for routine analysis. A recent review [9] described the reported analytical methods for the assessment and monitoring these drugs, alone or in combination. This indicating the proposed method considers the most sensitive method than other reported spectrophotometric methods.

### Analytical methods

#### iso-absorptive *point method*

**Fig. 3:**
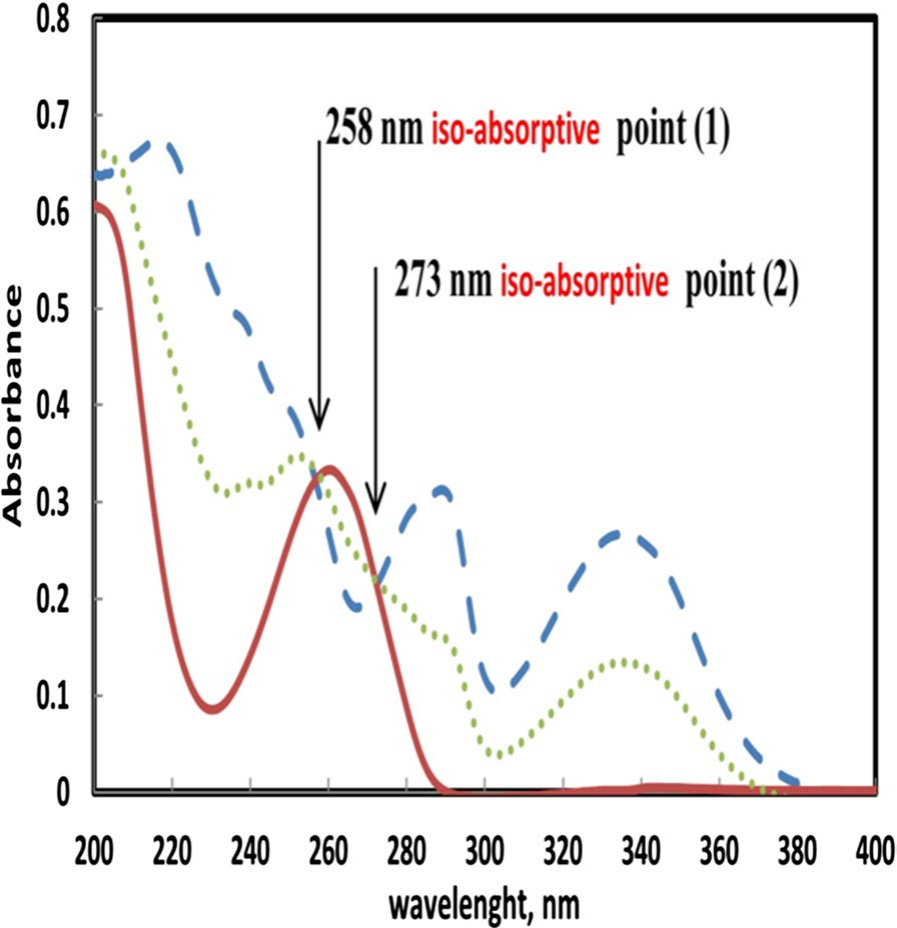
Zero order spectra with two points of intersection of (—) SOF 20.0 µg mL^−1^, (– – –) SMV 20.0 µg mL^−1^ and (⋯⋯) mixture containing (10.0 µg mL^−1^) of each drug

Upon examining the absorption spectra of equal concentration of SOF and SMV, spectral overlap is observed between 200 and 290 nm and the two spectra intersect at two wavelengths (iso-absorptive points: 258 and 273 nm). In this method, 273 nm was chosen after careful consideration since it provided more accurate results judged by % recoveries obtained. An experimental confirmation of the iso-absorptive point was attained by examining the absorbance of 20.0 µg mL^−1^ of SOF, 20.0 µg mL^−1^ of SMV and a mixture of 10.0 µg mL^−1^ of SOF and 10.0 µg mL^−1^ of SMV (Fig. 3). In this figure, SMV spectrum has multiple humps at 220, 290 and 340 nm but SOF has only one hump at 259 nm. After measuring the mixture’s spectrum, the hump of SMV at 220 was missing due to the summation of two curve and the SOF has low absorbance at 220 nm in comparison with SMV at this wavelength. In addition, the hump of SOF at 290 nm is mixed with the hump of SMV at 260 nm resulting in shoulder extended from about 250 nm to 290 nm. In all three cases, the absorbance value was the same at the iso-absorptive point. SMV could be directly determined using the mixture’s absorbance at 335 nm (SMV λ_max_) where SOF doesn’t interfere.

#### Ratio subtraction method

**Fig. 4:**
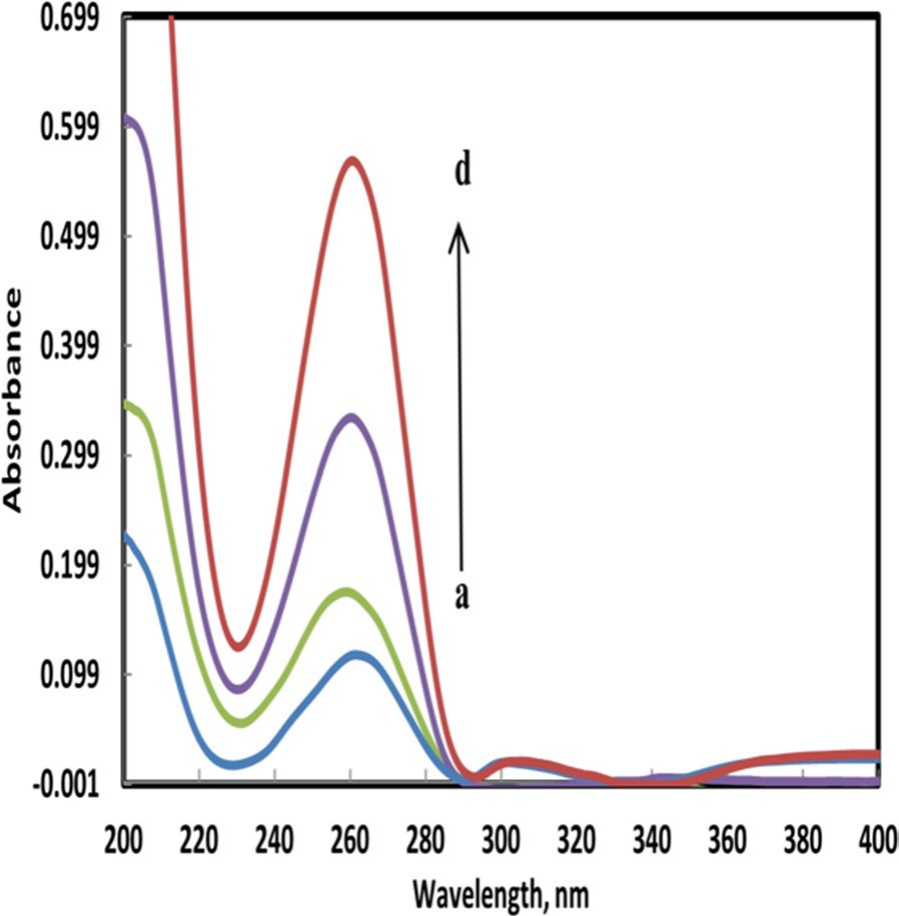
The resolved spectrum of SOF in the mixture from (a → d) at λ_max_ 260 nm in different concentration (5.0, 10.0, 20.0 and 30.0 µgmL^−1^, respectively) by using ratio subtraction method

After recording the mixture’s spectrum (200—400 nm), it was divided by the spectrum of 20.0 µg mL^−1^ SMV (B`). The resulting spectrum (*Spec 1*) represents $\frac{{\varvec{S}}{\varvec{O}}{\varvec{F}}}{{\varvec{S}}{\varvec{M}}{\varvec{V}}}+{\varvec{c}}{\varvec{o}}{\varvec{n}}{\varvec{s}}{\varvec{t}}{\varvec{a}}{\varvec{n}}{\varvec{t}}$. The constant is the absorbance plateau between 325–345 nm and after subtracting the ratio spectrum of SOF/SMV (*Spec 2*) was obtained. A simple multiplication of the ratio spectrum (*Spec 2*) by B` resolved SOF original spectrum that was part of the mixture. SOF can now be determined from the resolved spectrum at its λ_max_ of 260 nm using previously constructed calibration graphs. SMV can be directly determined from the mixture’s spectrum at its λ_max_ of 335 nm at which SOF had no absorbance. As shown in Fig. 4, the obtained spectrum of SOF extracted from the spectrum of the mixture using ratio subtraction method was superimposed with the spectrum of the pure SOF although there is very slight drift in the region above 340 nm.

#### Dual wavelength method

**Fig. 5:**
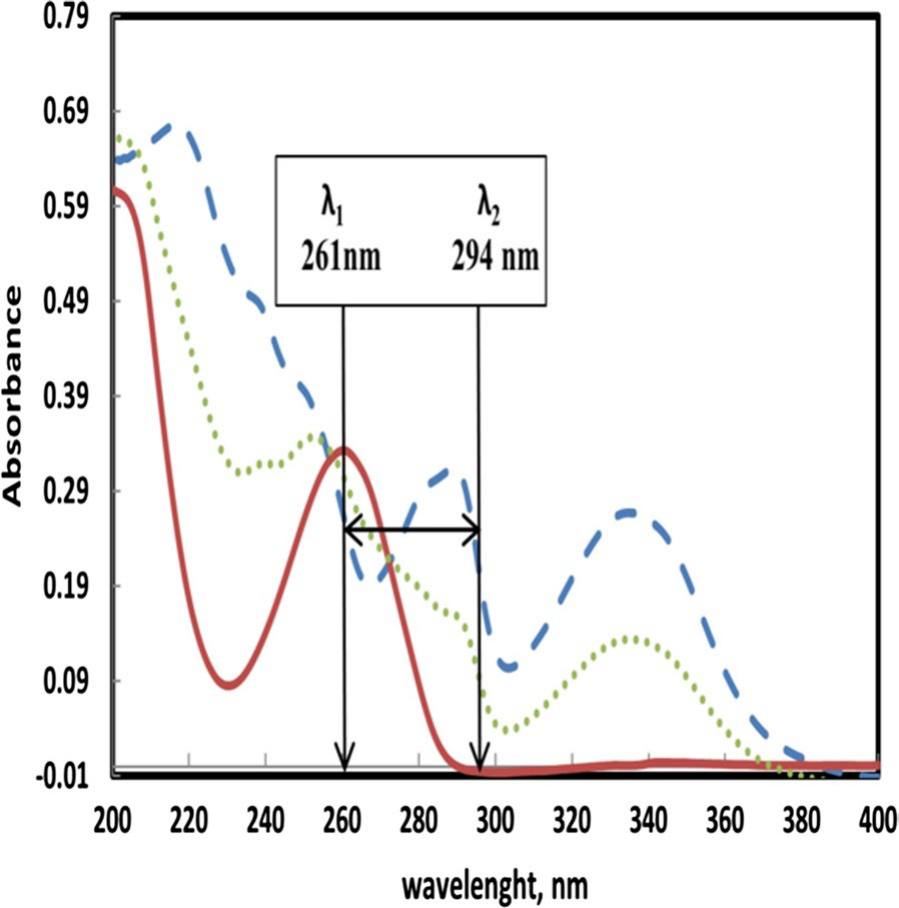
Zero order spectra of (—) SOF 20.0 µg mL^−1^, (– – –) SMV 20.0 µg mL^−1^ and (⋯⋯) mixture containing 10 µg mL.^−1^ of each drug, showing the two selected wavelengths (261 and 294 nm)

After thorough inspection of SOF and SMV spectra, two wavelengths emerged as best candidates for this method: 261 nm and 294 nm. At both wavelengths SMV absorbance was the same (ΔA_SMV_ = zero) while SOF absorbance was different, and this difference was also directly and strongly correlated to SOF concentration. The next step was to construct a linear calibration graph using pure SOF solutions and their corresponding difference in absorbance at both wavelengths (A_261 nm_–A_294 nm_). This calibration graph was used to directly find SOF concentration in the mixture. SMV concentration in the mixture could be directly found from its absorbance at 335 nm, where SOF doesn’t interfere (Fig. 5).

### Validation of the proposed methods

ICH guidelines regarding linearity, accuracy, precision, limit of detection and limit of quantitation [17] were followed to validate the methods presented in this work.

#### Linearity

Absorbance values at 335 nm were plotted against corresponding SMV concentration to construct its calibration curve. The linear regression equation’s terms were calculated where correlation coefficient was 0.9999 (Table 1). The linearity range was 3.0–50.0 µg mL^−1^ with LOD as low as 0.47 μg mL^−1^ Additional file 1: Figure S3.

**Table 1:** Analytical performance data for the calibration by using different methods for determination of SOF with SMV

Parameter	SMV (335 nm)	SOF		
		Method (I)	Method (II)	Method (III)
Linear range (µg mL^−1^)	3.0–50.0	2.0–50.0	2.0–50.0	4.0–50.0
Slope	0.0150	0.0114	0.0179	0.0179
Intercept	0.0277	0.0893	0.0605	0.0173
Standard deviation of intercept	0.0021	0.0021	0.0028	0.0029
Correlation Coefficient (r)	0.9999	0.9998	0.9998	0.9999
LOD (µg mL^−1^)	0.47	0.60	0.53	0.54
LOQ (µg mL^−1^)	1.44	1.84	1.60	1.64

*Method I:* The same manipulation was done for SOF but at 273 nm (iso-absorptive point). The linear regression equation’s terms listed in Table 1 show a correlation coefficient of 0.9998 over a linear range between 2.0 and 50.0 µg mL^−1^ with LOD of 0.60 μg mL^−1^ Additional file 1: Figure S4.

*Method II:* SOF absorbance values at 260 nm were used to construct its calibration graph and compute its linear regression equation. A correlation coefficient of 0.9998 over a concentration range of 2.0–50.0 µg mL^−1^was attained (Table 1) with a similar LOD of 0.53 μg mL^−1^ Additional file 1: Figure S5.

*Method III:* SOF ΔA values (A_261 nm_–A_294 nm_) were found to be strongly correlated (r = 0.9998) to their corresponding concentrations over a range of 4.0–50.0 µg mL^−1^ through linear regression analysis (Table 1). LOD was calculated as 0.54 μg mL^−1^ which is similar to method II Additional file 1: Figure S6. However, LOD for method I (iso-absorption point method) is slightly higher because the slope of the calibration curve for this method is lower than that in the two other methods. The reason for that is the value of absorbance for SOF at iso-absorption point is lower than that at its λ_max_ which was used in the two other methods.

#### Accuracy

Laboratory prepared mixtures of SMV and SOF at different known concentrations were used to assess the developed methods’ accuracy. For each mixture the new methods were applied, the absorbance values were recorded and employed into the corresponding linear regression equation (Table 1) to calculate the relevant drug’s concentration. The percent of the calculated concentrations to their true known counterparts (% recoveries) were calculated (Table 2) and found to have a mean close to 100% with RSD around 1%, which indicate the method’s high level of accuracy.

**Table 2:** Determination of SOF and SMV concentration in laboratory mixtures by the different proposed methods

Mix	Ratio	% Recovery^a^			
		SMV (335 nm)	SOF		
			Method I	Method II	Method III
1	1:1	100.56	101.66	100.10	102.21
2	1: 2	99.21	102.67	101.33	99.67
3	2:1	101.56	99.98	99.78	100.18
4	1:3	100.83	99.82	100.89	101.34
5	3:1	99.69	98.92	99.69	102.06
6	2:3	101.55	100.72	101.46	101.35
7	3:2	101.94	99.69	100.55	100.96
Mean		100.76	100.49	100.54	101.11
SD		1.02	1.29	0.71	0.92
% RSD		1.01	1.28	0.71	0.93

^a^the value is the mean of three determinations

#### Precision

Two levels of precision were assessed: intra- and inter-day. This was achieved by applying the proposed methods in three replicates of three different laboratory prepared mixtures of SMV and SOF. The analysis was performed in the same day at three different times (intra-day) and over three different days (inter-day). The percent recoveries and their RSD were calculated and found to be ranging between 98.54 and 102.81% with RSD almost always ≤ 2% (Table 3) proving the high level of precision of the proposed methods. Although baseline optimization for instrument was carried out for each measurement, the standard deviations in all methods for 7.5:20 mixture are relatively higher for the inter-day compered to intra-day. This may be due to the evaporation of the solvent during analysis. But this higher value of standard deviations is still within the acceptable value of precision according to ICH guideline.

**Table 3:** Intra- and inter- day precisions for the analysis of SOF and SMV in three laboratory mixtures by the proposed methods

Concentration (μg mL^−1^)		% Recovery^a^ ± RSD			
		Intra-day precision		Inter-day precision	
SMV	SOF	SMV^#^	SOF	SMV^#^	SOF
iso-absorptive point method					
7.5	20.0	100.39 ± 1.33	100.75 ± 1.13	100.18 ± 2.60	99.61 ± 2.44
10.0	20.0	99.90 ± 1.06	102.37 ± 2.00	102.81 ± 1.47	102.70 ± 0.53
20.0	10.0	102.39 ± 1.91	100.92 ± 0.96	99.83 ± 1.52	100.99 ± 2.00
Ratio subtraction method					
7.5	20.0	98.66 ± 0.75	101.88 ± 1.63	100.97 ± 1.15	99.32 ± 1.65
10.0	20.0	101.03 ± 1.07	100.27 ± 0.69	100.85 ± 1.67	101.64 ± 0.98
20.0	10.0	100.92 ± 1.45	99.75 ± 1.48	99.47 ± 0.85	102.45 ± 1.37
Dual wavelength method					
7.5	20.0	100.93 ± 0.65	102.42 ± 0.99	100.36 ± 1.26	99.32 ± 1.95
10.0	20.0	97.67 ± 1.17	100.83 ± 1.85	97.50 ± 1.17	99.64 ± 0.78
20.0	10.0	98.54 ± 1.83	100.26 ± 1.42	101.97 ± 1.59	102.53 ± 1.07

^a^The value is the mean of three determinations

^#^SMV was determined by direct determination suing the absorbance at 335 nm in all methods

### Analysis of the pharmaceutical dosage form

The methods presented in this work were utilized for quantitation of SMV and SOF in their pharmaceutical formulation (Merospevir^®^ capsules and Sofolanork^®^ tablets) laboratory made mixture. In order to compare the ability of the proposed methods for the determination of SMV and SOF in pharmaceutical preparation, the results obtained by applying each of the proposed methods and the reported TLC-Spectro-Densitometric methods [18] using t- and F-tests. The comparison revealed no significant differences at 95% confidence level (Table 4). Selectivity of the method was examined by studying the effect of the possible interference due to the presence of the common tablet excipients which used as coating and core for tablet such as, titanium dioxide, lactose monohydrate, magnesium stearate, and talc. Different mixtures containing different excipients in ratios similar to those present in the pharmaceutical formulations were prepared and analyzed by the proposed procedure. Results presented in (Table 5) show that the presence of either of these excipients did not significantly the results of the method as the % recovery values are close to 100%.

**Table 4:** Determination of dosage form in laboratory synthetic mixture of studied drugs and comparison with reported TLC-Spectro-Densitometric method

Method	% Recovery^a^ ± SD	
	SOF	SMV
iso-absorptive point	102.12 ± 1.21 (t = 1.91, F = 1.88)^b^	
Ratio subtraction	99.83 ± 1.78 (t = 1.56, F = 3.67)	100.53 ± 1.78^c^ (t = 0.98, F = 2.55)
Dual wavelength	100.78 ± 1.88 (t = 1.46, F = 4. 77)	
Reported methods^18^	100.01 ± 1.52	99.47 ± 1.39

^a^the value is the average of five measurements for both the proposed and reported methods

^b^the values in parentheses are t- value and F- value. Tabulated values at 95% confidence limit are t = 2.306 and F = 6.338

^c^SMV was determined by measuring the absorbance at λ 335 nm in all methods

**Table 5:** Analysis of pure studied drugs in presence of some common tablet and capsule excipients (1.0 mg mL^−1^) using the proposed methods

Excipients	% Recovery ± SD*			
	SMV (335 nm)	SOF		
		Method I	Method II	Method III
Titanium dioxide	100.63 ± 0.89	99.63 ± 0.99	102.09 ± 0.54	98.89 ± 1.25
Lactose monohydrate	101.16 ± 1.79	100.16 ± 1.55	100.57 ± 1.93	100.50 ± .1.71
Magnesium stearate	99.88 ± 1.65	98.88 ± 2.00	101.98 ± 1.69	101.83 ± 0.63
Talc	99.87 ± 0.98	101.00 ± 1.78	99.09 ± 1.02	100.40 ± 1.33

^*^Mean value of three determinations, (SD) standard deviation

### The statistical comparison of mixture pure powder with that of reported method

In order to compare the ability of the proposed methods for the determination of SMV and SOF in pure powder, the results obtained by applying each of the proposed methods and the reported TLC-Spectro-Densitometric methods [18] using t- and F-tests. The comparison revealed no significant differences at 95% confidence level (Table 6).

**Table 6:** Determination of pure powder in laboratory synthetic mixture of studied drugs and comparison with reported method

Method	% Recovery^a^ ± SD	
	SOF	SMV ^c^
iso-absorptive point	99.63 ± 1.00 (t = 0.97, F = 1.58)^b^	
Ratio subtraction	100.16 ± 1.55 (t = 1.33, F = 2.55)	101.22 ± 1.55 (t = 1.98, F = 1.78)
Dual wavelength	101.98 ± 1.69 (t = 1.88, F = 3. 54)	
Reported methods^18^	100.01 ± 1.52	99.47 ± 1.39

^a^the value is the average of five measurements for both the proposed for pure drugs and reported methods

^b^the values in parentheses are t- value and F- value. Tabulated values at 95% confidence limit are t = 2.306 and F = 6.338

^c^SMV was determined by measuring the absorbance directly at λ 335 nm in all methods

### Greenness evaluation of the proposed system

Analysts have a lot of responsibility when it comes to protecting the environment and people from harmful chemicals and organic waste that are produced as a result of chemical and pharmaceutical activities [19, 20]. Green chemistry must be created and upgraded on a regular basis. To assess an analytical method's 'ecological worth,' recent considerations such as the analytical eco scale score and the Environmental Quality Methods Index marking have been utilized [21, 22]. In the present work, Eco-Scale Score was utilized to determine the greenness of the proposed system. An analytical eco-scale assessment result is a number that represents a penalty point deducted from a total of 100; it is a result obtained for 'ultimate green analysis.' These points highlight the risks that researchers face during the study process. The greener the analysis, the higher the score (indicated by a large number) [23]. The eco-scale score for the developed technique was 95 because there was no extraction step, no heating, and the energy-consuming procedure was less than 0.1 kW h per sample. Results in (Table 7) indicate that the present method was environmentally friendly.

**Table 7:** Penalty points calculated based on Eco Scale Score for the greenness evaluation of the present method

Item	Parameter	Word sign	PP score
Technique	Spectrophotometry	LSH	1
Reagent(s)	Non		0
Solvent	Ethanol (< 10 mL)	LSH	1
Heating	No heating		0
Temperature	Room temperature		0
Cooling	No cooling		0
Energy (kWh per sample)	< 10 mL		0
Waste	1–10 mL		3
Occupational hazards			0
(TPPs)			5
Eco-scale total score	= 100 − TPP		95

MSH is an abbreviation for the more severe hazard, LSH for the Less severe hazard, and TPPs for the Total penalty points

## Conclusion

This work was devoted to answer the challenge of accurate and precise simultaneous quantification of Sofosbuvir and Simeprevir without prior separation. There was an added challenge that the developed methods should venture away from chromatography and into the realm of spectrophotometry if they are to be a viable simple and cheap yet reliable choice. Simeprevir could directly be determined without interference from Sofosbuvir. The real challenge was to determine Sofosbuvir in such mixture because of the significant spectral overlap. Chemometric methods were therefore an obvious choice; that could resolve complex mixtures using a simple spectrophotometer with only mathematical manipulations. The investigated drugs’ mixtures were accurately and precisely analysed in bulk powders and pharmaceutical dosage form using iso-absorptive point, ratio subtraction and dual wavelength-based methods. The procedures were simple and quick as well as environmentally friendly as they don’t need a large volume of solvents. The proposed methods were validated and proved they could be efficiently utilized for the routine analysis of the studied analytes in quality control laboratories with acceptable accuracy and precision. In conclusion, the dual wavelength method is the best method for SOF determination because its sensitivity is higher than the iso-absorptivity method and its procedure is simpler than the ratio subtraction method.

## Supplementary Information

**Additional file 1: Figure S1.** UV absorption spectra of SOF and SMV showing the two iso-absorptive points. **Figure S2.** UV absorption spectra of SOF and SMV showing the dual wavelengths at which the difference I absorbance for SOF is maximum and for SMV equal zero. **Figure S3.** Calibration curve for the determination of SMV by the proposed method at 335 nm. **Figure S4.** Calibration curve for the determination of SOF by the Isosbestic point method. **Figure S5.** Calibration curve for the determination of SOF by the Ratio subtraction method. **Figure S6.** Calibration curve for the determination of SOF by Dual wavelength method.

## Data Availability

The data that support the findings of this study are available from the corresponding author upon reasonable request.
