# Supplementary material for: Mathematical processing of absorption as green smart spectrophotometric methods for concurrent assay of hepatitis C antiviral drugs, Sofosbuvir and Simeprevir: application to combined pharmaceutical dosage forms and evaluation of the method greenness
Source: BMC Chem. 2023 Jul 14;17(1):75. doi: 10.1186/s13065-023-00984-5 (PMC10347804; doi:10.1186/s13065-023-00984-5)
Supplement: Supplementary file 1 — Additional file 1: Figure S1. UV absorption spectra of SOF and SMV showing the two iso-absorptive points. Figure S2. UV absorption spectra of SOF and SMV showing the dual wavelengths at which the difference I absorbance for SOF is maximum and for SMV equal zero. Figure S3. Calibration curve for the determination of SMV by the proposed method at 335 nm. Figure S4. Calibration curve for the determination of SOF by the Isosbestic point method. Figure S5. Calibration curve for the determination of SOF by the Ratio subtraction method. Figure S6. Calibration curve for the determination of SOF by Dual wavelength method. [file 13065_2023_984_MOESM1_ESM.docx]

**Supplementary materials (S)**

**Mathematical Processing of Absorption as Green Smart Spectrophotometric Methods for Concurrent Assay of Hepatitis C Antiviral Drugs,** **Sofosbuvir and** **Simeprevir: Application to** **Combined Pharmaceutical Dosage Forms** **and** **evaluation of the method greenness.**

**Sayed M Derayea^1^****,** **Ahmed A Abu-hassan^2^, Afhmed A. Hamad^2^, Walid E. Eltoukhi^2^, Amal E. Hamad^3^, Bassam Shaaban Mohammed****^*3^**

**^1^** Department of Analytical Chemistry, Faculty of Pharmacy, Minia University, Minia 61519, Egypt.

^2^ Pharmaceutical Analytical Chemistry Department, Faculty of Pharmacy, Al-Azhar University, Assiut Branch, Assiut, Egypt.

**^3^** Department of Pharmaceutical Analytical Chemistry, Faculty of Pharmacy, Menoufia University, Shebin El‐Kom, Menoufia, Egypt.

^*^ Correspondence author: Bassam.shaaban@phrm.menofia.edu.eg


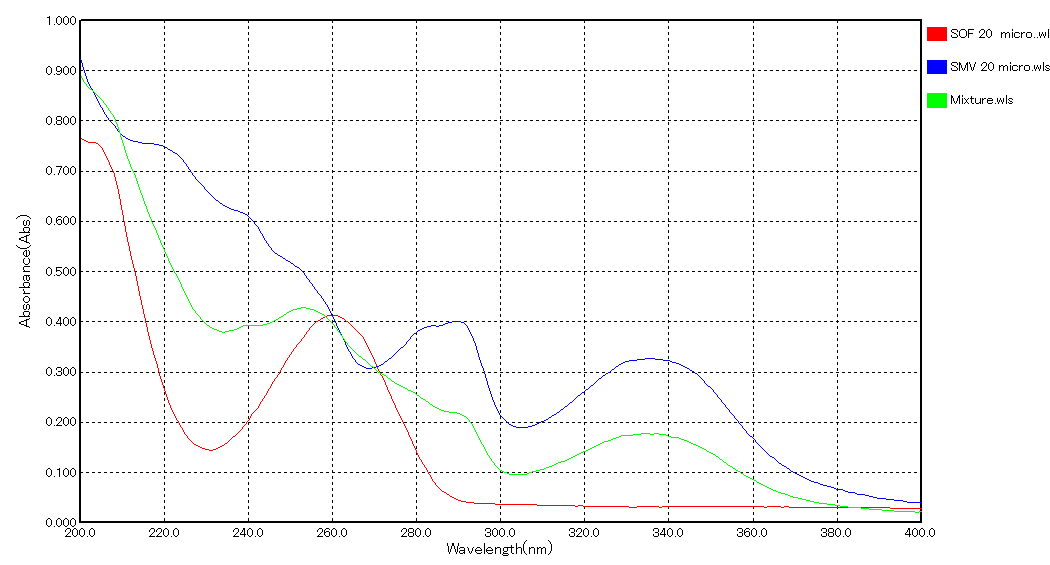


Iso-absorptive point at 258 nm

Iso-absorptive point at 273 nm

Fig. (S1): UV absorption spectra of SOF and SMV showing the two iso-absorptive points.


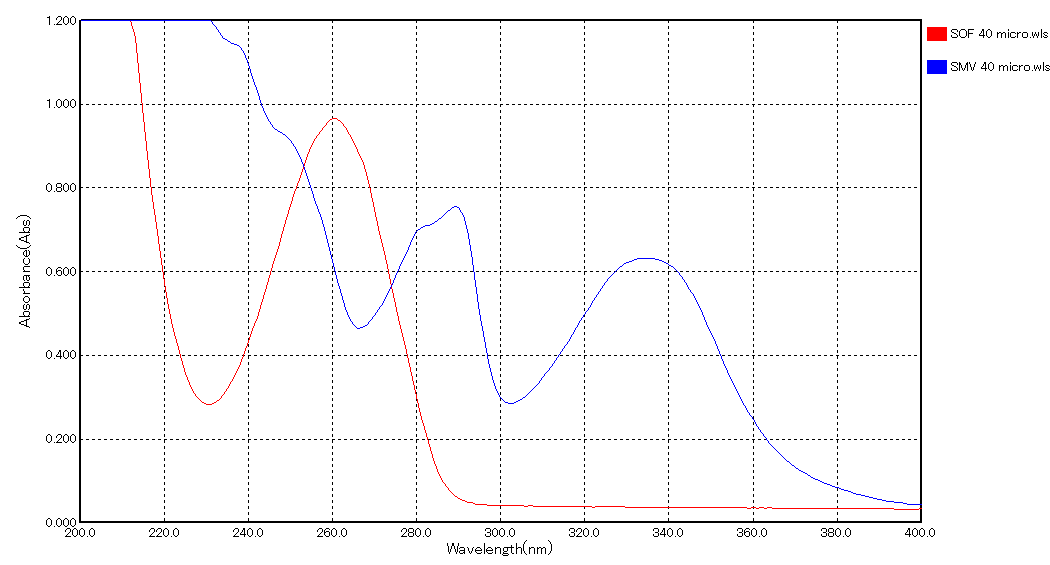


λ 294 nm

λ 258 nm

Fig. (S2): UV absorption spectra of SOF and SMV showing the dual wavelengths at which the difference I absorbance for SOF is maximum and for SMV equal zero.

Fig. (S3): Calibration curve for the determination of SMV by the proposed method at 335 nm.

Fig. (S4): Calibration curve for the determination of SOF by the Isosbestic point method.

Fig. (S5): Calibration curve for the determination of SOF by the Ratio subtraction method.

Fig. (S6): Calibration curve for the determination of SOF by Dual wavelength method.
